# SELF-PERCEPTION OF AGING AND THE SELF-REPORTED HEALTH/FRAILTY PARADOX

**DOI:** 10.1093/geroni/igad104.2105

**Published:** 2023-12-21

**Authors:** Brian Buta, Melissa Hladek, Mallak Alzahrani, Qianli Xue

**Affiliations:** Johns Hopkins University, Baltimore, Maryland, United States; Johns Hopkins University, Baltimore, Maryland, United States; Johns Hopkins University, Baltimore, Maryland, United States; Johns Hopkins, Baltimore, Maryland, United States

## Abstract

We aimed to study the cross-sectional association between self-perception of aging (SPA) and frailty paradox, defined as self-reporting excellent/very good health despite being frail or fair/poor health despite being robust. The study sample included 2,950 participants from the Health and Retirement Study (2008 wave) who were 65 years and older, completed the Psychosocial and Lifestyle Questionnaire, and had no missing data on the variables included in the models. Frailty was measured by the physical frailty phenotype. Self-reported health (SRH) was rated as either excellent, very good, good, fair, or poor. SPA was measured using an 8-item questionnaire spanning subjective well-being, positive affect, and life satisfaction; respondents rated their agreement with statements such as “Things keep getting worse as I get older” and “So far, I am satisfied with the way I am aging.” Negative statements were reverse-coded and the average of all items was calculated; higher scores indicated more positive SPA. 181 (6%) participants met the criteria for the frailty paradox: 54 reported being in excellent/very good health despite being classified as frail, while 127 reported fair/poor health despite being classified as robust. After adjusting for potential confounding variables, including age, sex, race, education, and comorbidity burden, we found that individuals with higher SPA scores had greater odds of excellent/very good SRH despite frailty. Specifically, the odds nearly doubled for every one unit increase in SPA score (p-value< 0.01). Positive self-perception of aging may be a potential interventional buffer against negative impact of physical frailty on self-assessment of overall health status.

